# T1K+: A Database for Benchmarking Color Texture Classification and Retrieval Methods

**DOI:** 10.3390/s21031010

**Published:** 2021-02-02

**Authors:** Claudio Cusano, Paolo Napoletano, Raimondo Schettini

**Affiliations:** 1 Department of Electrical, Computer and Biomedical Engineering, University of Pavia, Via Ferrata 1, 27100 Pavia, Italy; claudio.cusano@unipv.it; 2 Department of Informatics, Systems and Communication, University of Milan, Bicocca, Viale Sarca 336, 20126 Milan, Italy; raimondo.schettini@unimib.it

**Keywords:** texture recognition, texture retrieval, color and Texture, texture features, texture descriptors, color texture databases

## Abstract

In this paper we present T1K+, a very large, heterogeneous database of high-quality texture images acquired under variable conditions. T1K+ contains 1129 classes of textures ranging from natural subjects to food, textile samples, construction materials, etc. T1K+ allows the design of experiments especially aimed at understanding the specific issues related to texture classification and retrieval. To help the exploration of the database, all the 1129 classes are hierarchically organized in 5 thematic categories and 266 sub-categories. To complete our study, we present an evaluation of hand-crafted and learned visual descriptors in supervised texture classification tasks.

## 1. Introduction

Texture classification and retrieval are classic problems in computer vision that find applications in many important domains including medical imaging, industrial inspection, remote sensing, and so on. In the last decade, the shift to deep learning within the computer vision community made it possible to face these domains with very powerful models obtained by training large neural networks on very large databases of images [1].

This data hungry approach promotes the collection of larger and larger databases. The result is an increase in variability of content and imaging conditions, but at the price of a reduced control over the experimental conditions. In the field of texture recognition, this change manifested as a progressive switch from the experimentation with carefully acquired texture samples to the use of images randomly downloaded from the web.

Many recent texture databases include images that do not depict only the texture patterns, but also the context in which they are placed. As a result, they lack distinctive properties of textures such as the stationarity of the distribution of local features. It is not surprising, therefore, that the most accurate models for texture classification are those that also excel in general image recognition. In fact, the state of the art consists in using large convolutional neural networks trained for generic image recognition tasks like the ILSVRC challenge [1].

In this paper we present the T1K+ database, a large collection of high-quality texture images for benchmarking color texture classification and retrieval methods. T1K+ contains 1129 texture classes ranging from natural subjects to food, textile samples, construction materials, etc. To help the exploration of the database, all 1129 classes are hierarchically organized in 5 thematic categories and 266 sub-categories. Unlike many other large databases, T1K+ does include images taken from the web, but pictures that have been especially acquired to be part of the collection. The acquisition protocol ensures that only images actually representing textures were collected, excluding images depicting scenes, objects, or other kinds of content. Thanks to these properties, the database allows the design of experiments especially aimed at understanding the specific issues related to texture classification and retrieval. Some of these experiments, together with their outcomes, are described in this paper.

The rest of the paper is organized as follows. Section 2 presents the main characteristics of the proposed database: acquisition conditions, type of classes, hierarchical organization of the classes, and distribution of the perceptual features. In this section, we also discuss how T1K+ compares with respect to existing texture databases. In Section 3, we present all the visual descriptors used in the evaluation while in Section 4 we present all the experiments performed. Section 5 discusses the results we obtained and Section 6 presents future challenges and conclusions.

## 2. The Database

The T1K+ database was conceived as a large collection of images of surfaces and materials. At the present time, the database includes pictures of 1129 different classes of textures ranging from natural subjects to food, textile samples, construction materials, etc. Expansions are planned for the future. The database is designed for instance-level texture recognition. That is, each surface is considered as having a texture of its own class. This feature differentiates the database from other large-scale collections of textures where classes are defined to contain multiple instances of the same concepts.

Images have been collected by contributors who were asked to take pictures of as many texture samples they could find during their daily activities. To do so they used their personal smartphones as acquisition devices. Modern smartphone cameras, in fact, are able to provide high-resolution pictures of reasonable quality, without sacrificing the ease of the acquisition procedure.

**Figure 1:**
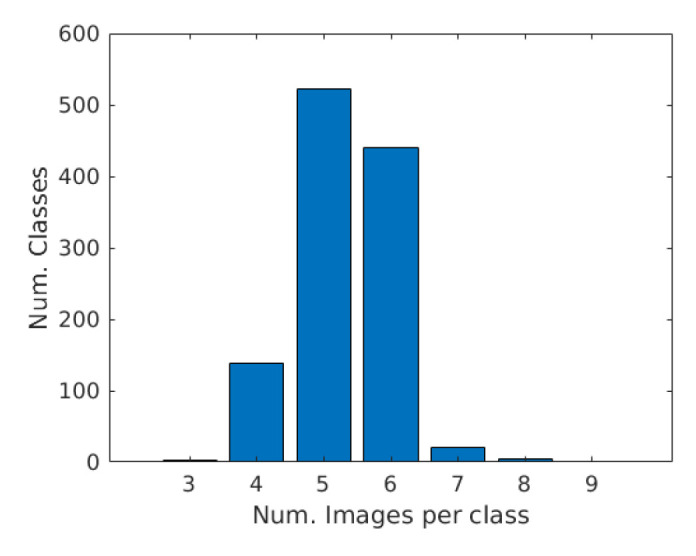
Distribution of the classes within the T1K+ database.

For each texture sample several pictures have been taken, each time slightly varying the viewpoint or the portion of the surface acquired. The contributors were also asked to vary the lighting conditions when possible, for instance by turning on and off artificial light sources (for indoor acquisitions) or by moving the sample in different positions (for movable texture surfaces). Pictures have been post-processed by excluding those with low overall quality (excessive motion blur was the main cause) and by removing near duplicates. In the end, a minimum of four images per texture sample were retained. The result was the collection of 6003 images in total, for an average of 5.32 images per class. Figure 1 shows the distribution of the classes over the data set images. Seeing the plot, we decided to not address the unbalancing in the class distribution. Unbalanced classes could become an issue in the future after the expansion of the database. In such a case, appropriate techniques such as those in [2,3,4,5] should be considered.

**Figure 2:**
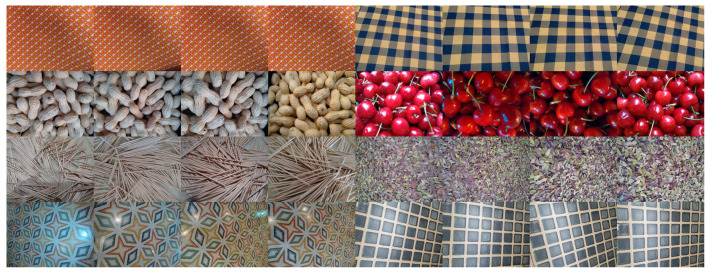
Samples of eight classes of textures in the T1K+ database.

All images are in the sRGB color space and have an average resolution of 2465×3312 pixels, making it possible to extract multiple non-overlapping patches at various scales. This way the data set can be used for different kind of experiments, from retrieval to classification. Figure 2 shows the images acquired for a selection of eight classes. Note the intra-class variability caused by the changes in the viewpoint and in the lighting conditions.

### 2.1. Composition

**Figure 3:**
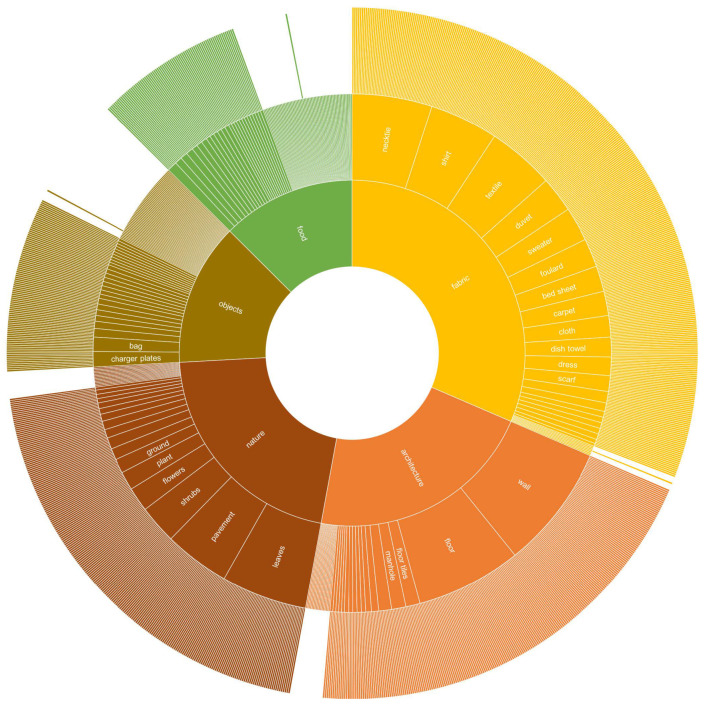
Hierarchical representation of T1K+ texture classes. The first ring is composed of five classes: nature, architecture, fabric, food, and objects. The second ring is composed of 266 classes: wall, floor, shirt, flowers, pavement, textile, and many others. The last ring is composed of 1129 leaves.

In order to better understand the composition of the database, we divided the 1129 classes in five thematic categories: nature (239 classes), architecture (241), fabric (355), food (143), and objects (151). These can be further subdivided into a second level of 266 categories and a third one of 1129 (in which there is one class per texture instance). Figure 3 shows the hierarchical organization of the three levels. Within this organization the database can be used to analyze classification methods in coarse- and fine-grained scenarios.

**Figure 4:**
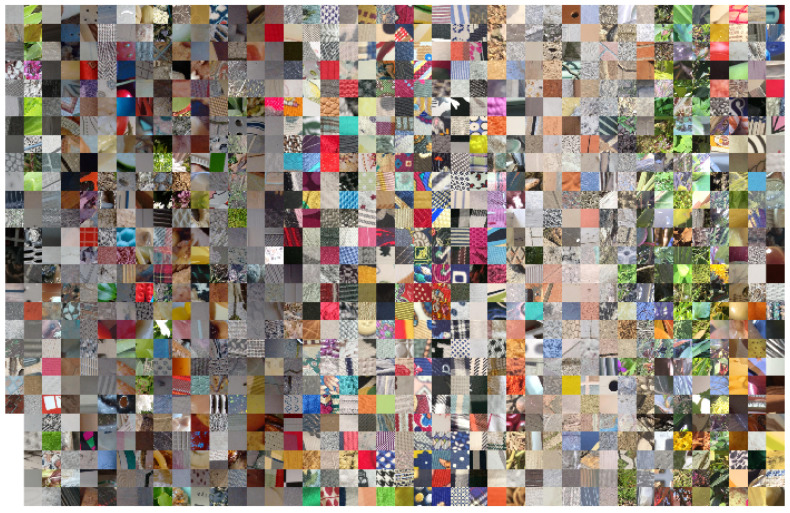
Overview of the database. For each class is included a patch taken from one of the images.

An overview of the inter-class variability in the database is given by Figure 4 which shows one sample for each of the 1129 classes. Note how there are clusters of classes with a similar dominant color (e.g., foliage), others with a high chromatic variability (e.g., fabrics). There are many regular and irregular textures, low- and high-contrast, etc. Overall, a large number of combination of possible texture attributes is represented in the database.

**Figure 5:**
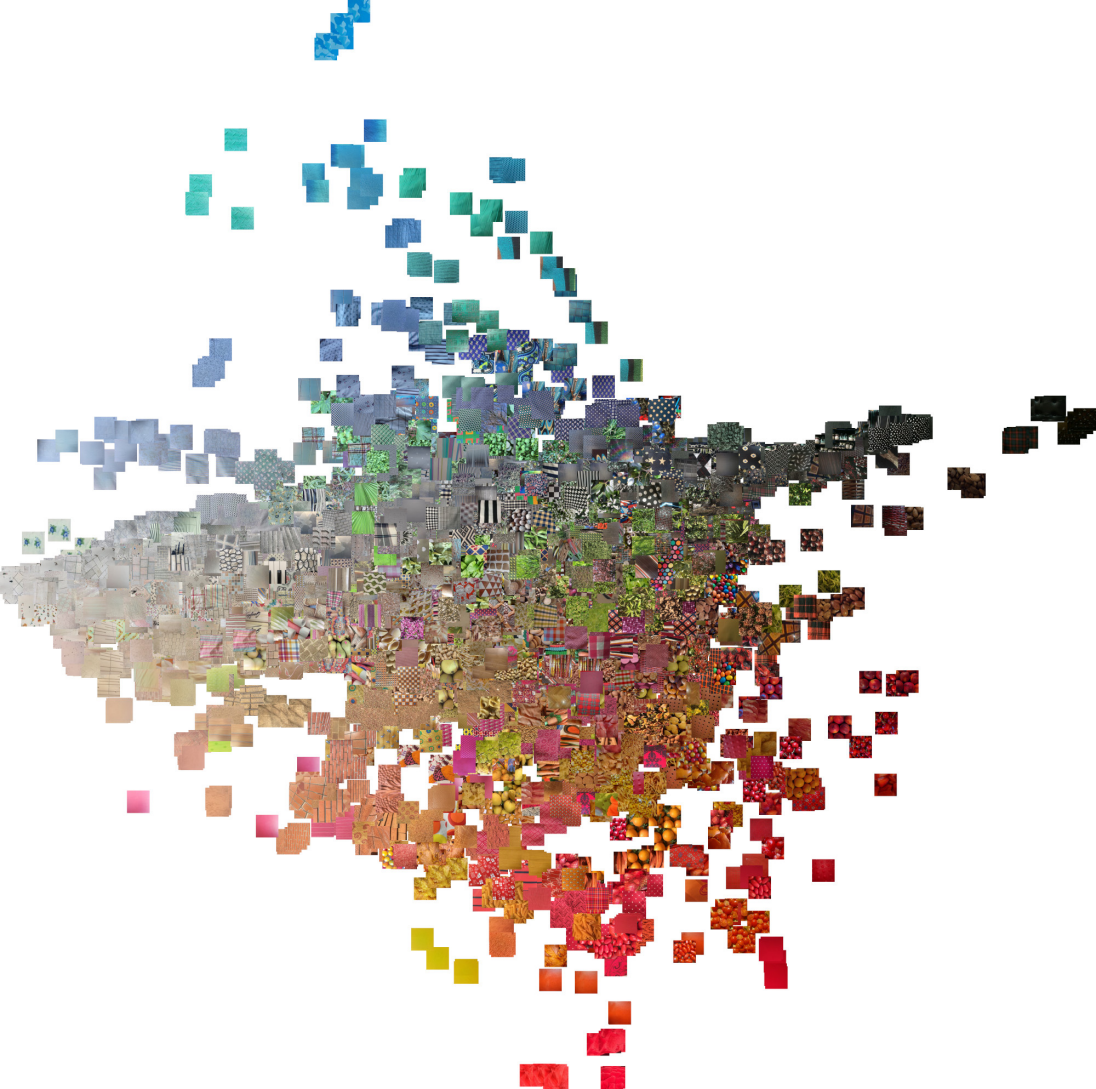
Result of the application of t-SNE to the image pixels.

**Figure 6:**
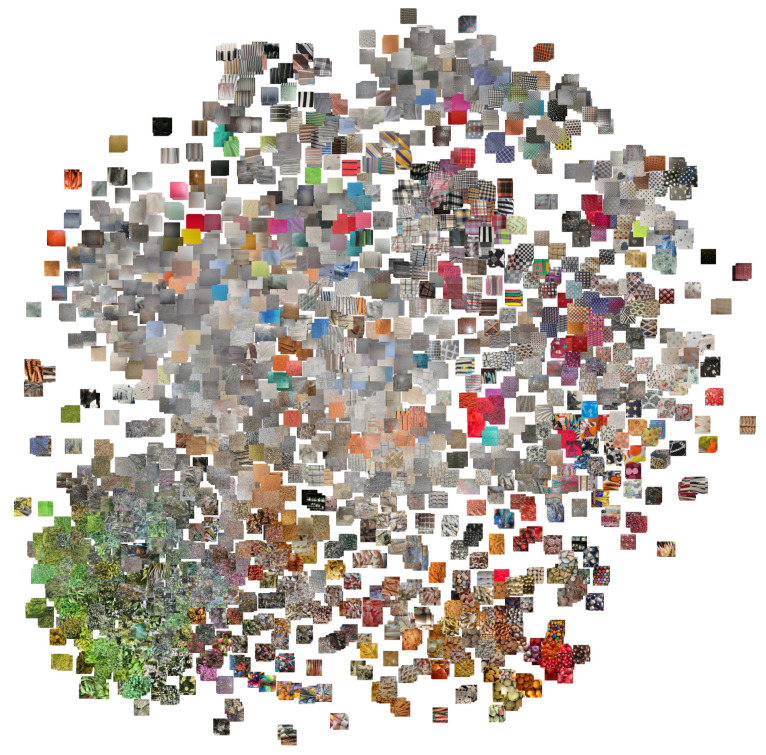
Result of the application of t-SNE to the features extracted by a Resnet50 CNN trained on ILSVRC data.

To better analyze the distribution of the content of the database we applied the t-SNE algorithm [6]. We run the algorithm twice, by directly using the image pixels as features and by using the features extracted by a Resnet50 convolutional network trained for image recognition on the ILSVRC training set. The algorithm places similar images near each other on the two-dimensional plane. The result can be observed in Figure 5 and Figure 6.

### 2.2. Perceptual Features Distribution

To further illustrate the main characteristics of our database, in this section we show statistics of perception-based features introduced by Tamura et al. [7] in 1978. These features are based on a set of psychological experiments which have the aim to assess how humans perceive texture properties like coarseness, contrast, directionality, line-likeliness, and roughness.

Coarseness is defined on the basis of the size of the texture elements. The higher the size, the coarser the texture and the smaller the size, the finer the texture. Contrast depends on the distribution (histogram) of gray-levels, the sharpness of edges, and the period of repeating patterns. Directionality is related to the probability that the variation of the pixels’ intensities occurs along certain predefined orientations. The higher are parallel lines within an image and the higher is the value of directionality. Line-likeliness is a property that encodes how much an image is perceived as composed by lines. Roughness is related to how surface is perceived by a haptic touch. The term ‘rough’ stands for a surface marked by protuberances, ridges, and valleys. The higher the presence of surface irregularities, the higher the roughness.

There are several implementations of these features, we refer to the one by Bianconi et al. [8] in which, differently from the original definition, each feature is represented by a real number in the [0, 1] interval. To better explore the statistics of this features, we divide the [0, 1] range into 5 bins and we plot for each features histograms over those 5 bins.

**Figure 7:**
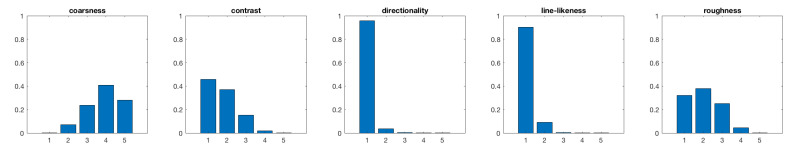
Distribution of the perceptual features of the T1K+database.

Figure 7 shows the histograms of the Tamura’s features of the T1K+database. Note that there is a good variability in terms of contrast, roughness, and coarseness. Directionality and line-likeness, instead, are generally low. In fact their value is typically high for artificially generated texture images.

### 2.3. Comparison with State-of-The-Art Texture Databases

Texture databases play a fundamental role in the development of texture analysis methods. Earlier databases, such as Brodatz [9] and VisTex [10], were formed by a single image per class. They were mainly used to define texture classification tasks, in which multiple patches were extracted and used as samples of the class corresponding to the image they were taken from. This approach was limited by the lack of intra-class variability, and it was abandoned in favor of databases in which the texture samples were taken under a multitude of acquisition conditions obtained by changing the viewpoint or the lighting conditions in a controlled way. The CUReT database is a notable example of this kind of database. It includes 61 texture samples, each one acquired under 93 different conditions. Other databases collected with the same approach are KTH-TIPS [11,12], ALOT [13], UIUC [14], RawFooT [15], and many others.

More recently, researchers departed from the traditional approach of taking controlled pictures of single surfaces and started to take pictures of texture images “in the wild”. Categories are also defined to increase the intra-class variability. Examples of this alternative approach are the DTD [16] and the FMD [17] databases.

Concerning the content of the images, most databases are generic and include a variety of surfaces. Some databases focus on specific domains; for instance, there are databases made of images of leaves [18], images of barks [19], food [15], ceramic tiles [20], terrain [21].

Table 1 summarizes the most relevant databases in the literature and compares them with the newly proposed T1K+. The database significantly increases the number of texture classes and the quality of the images in terms of resolution.

## 3. Benchmarking Texture Descriptors

We experiment with several state-of-the-art hand-crafted and learned descriptors [23]. For some of them we consider both color (RGB) and gray-scale (L) images, where L is defined as L = 0.299R + 0.587G + 0.114B. For all the feature vectors we consider the $l^{2}$ normalization. In the following we list the visual descriptors employed in this study:

- Hist L: this is a 256-dimensional gray-scale histogram [24];
- Hist RGB (with 256 bins) and 3 marg. hist.: these are two variants of RGB histograms, both of size 768 [25];
- Quantized RGB histogram (with 48 bins) [25];
- Spatial RGB histogram as described in the paper by Huang et al. [26]. Four subregions are considered;
- Chrom. Mom.: a feature vector composed of normalized chromaticity moments of size 10. We use the version defined by Pachos et al. [27];
- Segmentation-based Fractal Texture Analysis as described in the paper by Costa et al. [28] that outputs a 24-dimensional feature vector for a gray-level image;
- Cooccurrence matrix of color indexes as described in [29].
- Granulometry feature vector as described in [30,31]
- Gist: this feature vector is obtained considering eight orientations and four scales for each channel. The size is 1536 [32];
- DT-CWT: this is 24-dimensional Dual Tree Complex Wavelet Transform obtained by considering four scales, mean, and standard deviation, and three color channels. We use the implementation by Bianconi et al. [33,34];
- Color and Edge Directivity Descriptor (CEDD) is a 144-dimensional feature vector based on a fuzzy version of the five digital filters proposed by the MPEG-7 Edge Histogram Descriptor (EHD);
- Histogram of Oriented Gradients (HoG) is a 81-dimensional feature vector computed as nine histograms encoded with nine bins [35];
- Gabor: mean and standard deviation of six orientations extracted at four frequencies for each color channel filters. The final size is 96 [33,36];
- Local Binary Patterns (LBP) with a circular neighborhood of radius 2 and 16 elements, and 18 uniform and rotation invariant patterns for each channel for a total of 54 [37];
- Local Binary Patterns (LBP-nri) with a circular neighborhood of radius 2 and 16 elements, and 243 uniform and no-rotation invariant [37];
- LBP-nri combined with a 256-dimensional Local Color Contrast (LCC) [38,39,40,41,42];
- Learned descriptors, obtained as the intermediate representations of several Convolutional Neural Networks [43]: VGG 16 and 19, SqueezeNet, Inception V3, Google Net, Residual Network of depth 50 (ResNet-50). The resulting feature vector is obtained by removing the final softmax nonlinearity and the last fully-connected layer. The network used for feature extraction is pre-trained for scene and object recognition [44] on the ILSVRC-2015 dataset [45].

## 4. Experiments

The T1K+ database contains 1129 classes and an average of about 5.32 images for each class. We divide the database in two sets: 4871 training images (4 images per class on average) and 1129 test images (1 image per class). Each image is further separated in tiles of size 250×250. Following a chessboard strategy: we keep the tiles corresponding to the “black” cases on the chessboard and we discard the remaining ones. The resulting tiles are: 584,836 for training (500 per class) and 134,957 for test (100 per class). Figure 4 shows sample tiles, one for each class.

To alleviate the computational burden we reduce, for all the experiments, the training and test sets by a factor of 10 and 8, respectively, thus obtaining: 58,484 training tiles (50 per class) and 16,870 tiles (15 per class). Later on we will demonstrate that this choice does not influence the goodness of this study.

### 4.1. Evaluation Metrics

In all the experiments we measure performance in terms of:

(1) $$\begin{array}{cc} Accuracy & =\frac{\sum_{c=1}^{K}TP_{c}}{\sum_{c=1}^{K}P_{c}}, \end{array}$$

(2) $$\begin{array}{cc} Precision & =\frac{1}{K}\sum_{c=1}^{K}Pr_{c}=\frac{1}{K}\sum_{c=1}^{K}\frac{TP_{c}}{TP_{c}+FP_{c}}, \end{array}$$

(3) $$\begin{array}{cc} Recall & =\frac{1}{K}\sum_{c=1}^{K}Re_{c}=\frac{1}{K}\sum_{c=1}^{K}\frac{TP_{c}}{TP_{c}+FN_{c}}, \end{array}$$

(4) $$\begin{array}{cc} F1 & =\frac{1}{K}\sum_{c=1}^{K}F1_{c}=\frac{1}{K}\sum_{c=1}^{K}2\cdot \frac{Pr_{c}\cdot Re_{c}}{Pr_{c}+Re_{c}}, \end{array}$$

where, *K* is number of classes, *c* represents a generic class, $P_{c}$ is the number of positives for the class *c*, $TP_{c}$ is the number of true positives for the class *c*, $FP_{c}$ is the number of false positives for the class *c*, and $FN_{c}$ is the number of false negatives for the class *c*.

### 4.2. Texture Classification Experiments

The aim of the first experiment was to evaluate the robustness of the visual descriptors in a traditional classification task. We use a 1-Nearest Neighbor (1-NN) classifier with the Euclidean distance. We evaluate the visual descriptors on the three classification tasks, each for each semantic level depicted in Figure 3: 1129 classes, 266 classes, and 5 classes.

Table 2 shows results achieved by each visual descriptors in the 1129-classes problem. Residual Networks, both RGB and L, outperform by about 40% and 30%, respectively, the best hand-crafted descriptor that is the RGB histogram. The best accuracy is 82.34%.

Table 3 shows results achieved by each visual descriptors in the 266-classes problem. Additionally in this case, Residual Networks, both RGB and L, outperform hand-crafted descriptors. The best accuracy is 85.77%, that is about 3% higher than the accuracy of the 1129-classes experiment.

Table 4 shows results achieved by each visual descriptors in the 5-classes problem. Again, learned features outperform hand-crafted descriptors. The best accuracy is now very high: 93.41%. In this task hand-crafted descriptors achieve higher accuracy with respect to the previous experiments. The RGB histogram achieves an overall accuracy of 64.92%.

**Figure 8:**
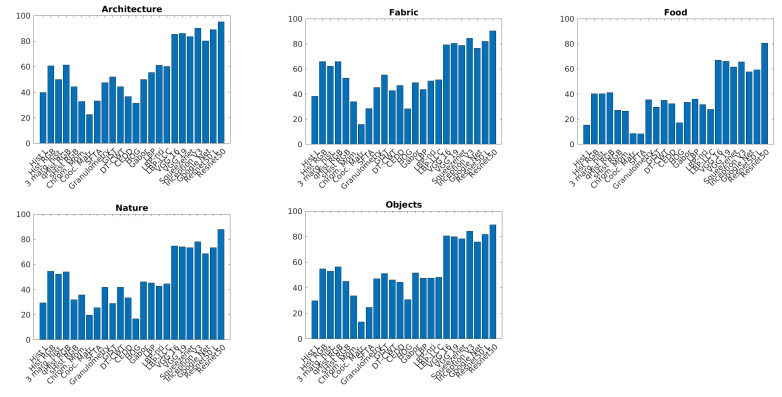
Visual descriptor comparison for each of the thematic categories.

**Figure 9:**
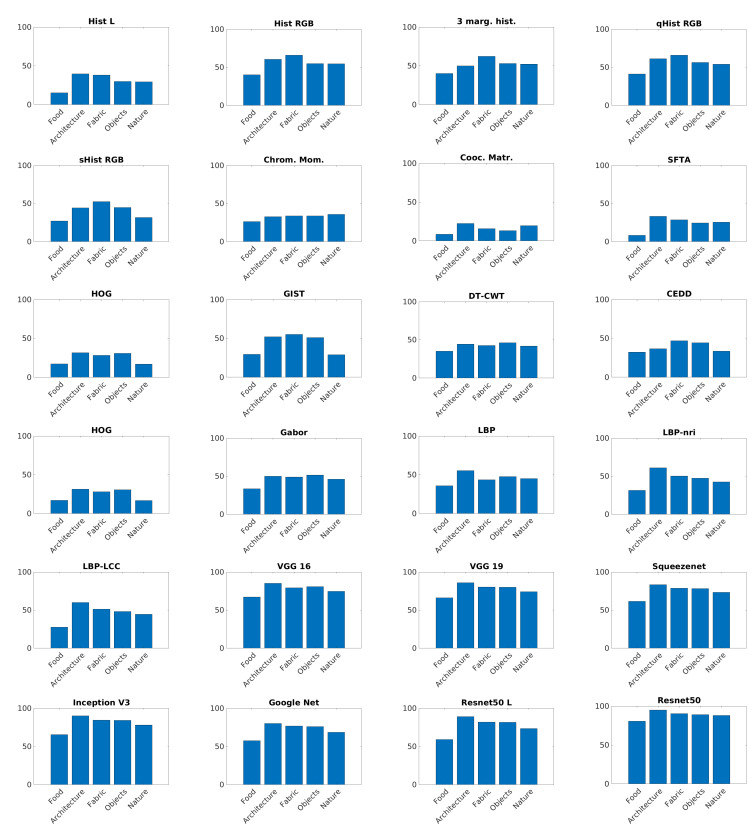
Performance of each visual descriptor on each thematic categories.

Figure 8 and Figure 9 show accuracy comparison of each texture descriptor with respect to the five thematic categories. In all the categories colored Residual Networks overcome other descriptors. Hand-crafted features achieve a lower accuracy with respect to other classes.

### 4.3. Accuracy vs. Training Set Size

**Figure 10:**
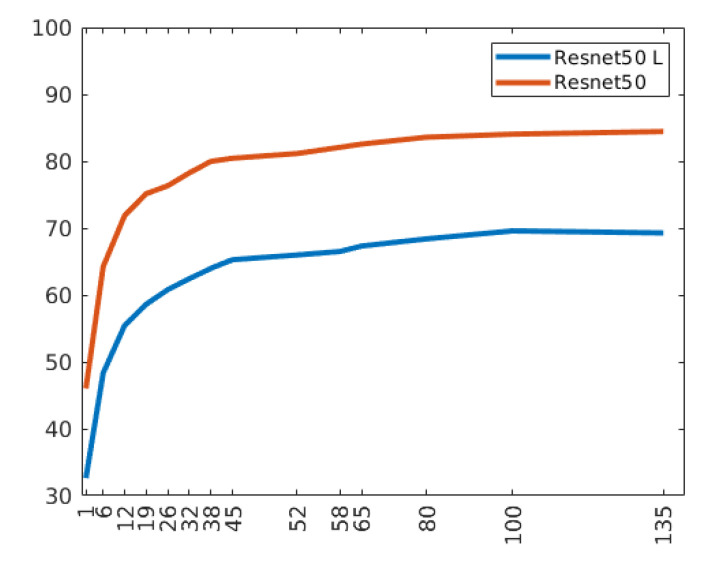
Accuracy trend as the average number of tiles per class of the training set increases.

To demonstrate that the reduction of training samples does not influence the goodness of this study, we experiment different sizes of the training set ranging from 1 to 135 tiles on average for each class. Figure 10 shows the accuracy trend with respect to the increase in the number of training samples for each class. For this experiment, both the ResNet50 and ResNet50 L are employed. In both cases, the curve reaches a plateau after 45 tiles for each texture class. The number of tiles used in the texture experiments is 50 on average for each class.

### 4.4. One Shot Texture Classification

The aim of this experiment was to evaluate the goodness of visual descriptors when only one tile for each texture class is available. We employ one tile per class as the training samples and a total of 16,870 tiles as the testing samples. We repeat the experiment 10 times to reduce possible bias related to the choice of the single tile.

Table 5 shows results achieved on average by each visual descriptor. In this case, none of the descriptors achieve good performance. The best is still the ResNet-50 RGB with 43.48% of accuracy. Most of the hand-crafted descriptors are below 10% accuracy.

## 5. Discussion

The results of the experiments give important insights about the T1K+ database. First of all, it is clear that features computed by pre-trained convolutional networks vastly outperform hand-crafted descriptors. This fact confirms the large amount of evidence that characterized the last decade of research in computer vision. Complex models are definitely more effective in automatically identifying discriminative visual representations than any human expert. Among neural architectures, Resnet50 clearly outperformed all the alternatives considered in all the tasks.

Another interesting result is that color is still a very important cue for the recognition of textures. Descriptors that make use of it tend to perform better than their color-blind versions. This also applies to features extracted by neural networks. Despite the variability in the lighting conditions, color information allows to distinguish most textures, leaving as ambiguous only the texture classes with similar color distributions. In fact, with color histograms, we obtained the best results among hand-crafted descriptors. This contradicts other results in the literature, and shows that increasing the number of classes and, therefore, increasing their density in the feature space seriously hampers even complex hand-crafted descriptors such as HOG, GIST, and LBP.

A very important factor in obtaining high classification accuracy is the availability of a large number of training samples. Without it, the descriptors alone cannot capture the intra-class variability. The extreme case is the one-shot learning scenario, in which we observed relatively poor results with all the descriptor. The highest accuracy in that scenario has been obtained by neural features and it is only of about 43%. This result shows that there is still plenty of room for possible improvement in this field.

## 6. Conclusions

In this paper we presented T1K+, a database of high-quality texture images featuring 1129 diverse classes. Intra-class variability is ensured by the acquisition protocol, which required changes in the viewpoint and in the lighting conditions across multiple acquisitions of the same texture. The database is publicly available at http://www.ivl.disco.unimib.it/activities/t1k/ and we plan to continue the collection of new texture classes to further enlarge it.

The database is not only large but is also organized in a taxonomy that makes it suitable for a large variety of experiments and investigations ranging from classification to retrieval. To provide an initial benchmark, we tested several well known texture descriptors for classification using nearest neighbor classifiers.

Several further investigations are suggested:

- The database has been acquired with a weak control of viewing and lighting conditions. We have shown in [38,46] that different color temperature light can be artificially simulated and that these may have an impact on texture classification performance. It would be interesting to generate such an augmented database and verify if the absolute and relative ranking of the descriptor performance is maintained.
- Other type of image artifacts and/or distortions could be artificially generated on the T1K+, such as noise, blur, jpeg compression, etc. In that case, the performance of the CNNs may decrease.
- Deep learning models could be especially designed for texture classification, with the aim of making it possible to obtain a high classification accuracy without relying on large models pre-trained for object and scene recognition.
- It would be also interesting to focus on specific portions of the T1K+, such as architecture, food, leaves, textile, etc. and to develop optimized ad-hoc methods for each domain.

## Figures and Tables

**Table 1:** Summary of a selection of texture databases in the literature. All the databases except Brodatz contain color images.

Acronym	Subject	Classes	Images Per Class	Image Size	Year	Reference
Brodatz	Mixed	111	1	640×480	1966	[9]
VisTex	Mixed	167	1	785×512	1995	[10]
CUReT	Mixed	61	93	200×200	1999	[22]
KTH-TIPS	Mixed	10	81	200×200	2004	[11]
UIUC	Mixed	25	40	640×480	2005	[14]
KTH-TIPS2b	Mixed	11	432	200×200	2006	[12]
V×C_TSG	Ceramic tiles	42	12	128×128	2008	[20]
ALOT	Mixed	250	100	1536×1024	2009	[13]
FMD	Materials	10	100	512×384	2009	[17]
PlantLeaves	Plant leaves	20	60	128×128	2009	[18]
DTD	Texture attributes	47	120	640×640	2014	[16]
NewBarkTex	Barks	6	273	64×64	2014	[19]
RawFooT	Food	68	46	800×800	2016	[15]
GTOS	Terrain	40	856	240×240	2016	[21]
T1K+	Mixed	1129	5.3	2465×3312	2021	This paper

**Table 2:** Benchmark using 1-Nearest-Neighbor (1-NN) (1129 classes).

Features	Acc.	Pr	Re	F1
Hist L	16.48	14.73	16.52	15.58
Hist RGB	42.24	41.97	42.38	42.18
3 marg. hist.	37.67	36.56	37.98	37.25
qHist RGB	42.49	42.10	42.62	42.36
sHist RGB	26.28	31.12	26.63	28.70
Chrom. Mom.	15.70	15.94	15.88	15.91
Cooc. Matr.	4.55	4.62	4.52	4.57
SFTA	10.28	10.03	10.35	10.19
Granulometry	26.40	27.08	26.63	26.86
GIST	29.53	33.28	29.74	31.41
DT-CWT	25.20	25.44	25.33	25.39
CEDD	22.31	23.05	22.45	22.74
HOG	11.67	12.88	11.70	12.27
Gabor	29.63	30.01	29.84	29.92
LBP	26.94	27.51	26.93	27.21
LBP-nri	31.26	31.91	31.23	31.57
LBP-LCC	29.88	29.76	29.92	29.84
vgg16	64.85	65.84	64.79	65.31
vgg19	65.17	65.75	65.13	65.44
squeezenet	62.51	62.55	62.51	62.53
Inception V3	71.09	71.69	71.10	71.39
Google Net	58.40	58.71	58.36	58.53
Resnet50 L	67.23	67.53	67.17	67.35
Resnet50	82.34	82.95	82.32	82.64

**Table 3:** Benchmark using 1-NN (266 classes).

Features	Acc.	Pr	Re	F1
Hist L	21.62	13.69	14.79	14.22
Hist RGB	47.58	40.33	39.22	39.77
3 marg. hist.	42.86	33.44	35.22	34.30
qHist RGB	47.89	40.51	39.82	40.16
sHist RGB	31.61	31.82	23.60	27.10
Chrom. Mom.	21.75	16.39	16.85	16.62
Cooc. Matr.	9.31	4.80	4.76	4.78
SFTA	15.52	10.12	10.12	10.12
Granulometry	32.53	27.37	28.24	27.80
GIST	34.70	29.89	29.24	29.56
DT-CWT	31.71	26.47	27.56	27.00
CEDD	28.62	21.96	22.07	22.02
HOG	17.36	13.63	12.71	13.15
Gabor	36.39	31.33	31.15	31.24
LBP	33.87	29.82	29.98	29.90
LBP-nri	38.11	32.04	31.68	31.86
LBP-LCC	36.92	30.30	30.21	30.25
vgg16	70.63	68.01	66.96	67.48
vgg19	70.81	67.06	67.07	67.06
squeezenet	68.09	62.47	63.62	63.04
Inception V3	76.73	71.56	70.65	71.10
Google Net	64.23	59.70	59.39	59.55
Resnet50 L	72.32	65.57	65.40	65.49
Resnet50	85.77	82.78	82.13	82.45

**Table 4:** Benchmark using 1-NN (5 classes).

Features	Acc.	Pr	Re	F1
Hist L	42.36	38.96	38.06	38.50
Hist RGB	64.92	63.59	61.82	62.69
3 marg. hist.	62.54	60.19	59.94	60.07
qHist RGB	65.14	63.70	62.14	62.91
sHist RGB	52.77	54.80	47.55	50.92
Chrom. Mom.	47.26	44.96	45.32	45.14
Cooc. Matr.	33.79	32.48	32.60	32.54
SFTA	40.26	37.61	37.66	37.63
Granulometry	55.91	53.98	54.36	54.17
GIST	56.05	55.06	53.69	54.37
DT-CWT	56.82	54.81	54.85	54.83
CEDD	51.44	49.92	49.68	49.80
HOG	41.83	41.37	39.75	40.55
Gabor	61.03	59.03	58.98	59.00
LBP	59.99	57.87	57.86	57.86
LBP-nri	60.89	59.03	58.12	58.57
LBP-LCC	61.01	58.08	58.08	58.08
vgg16	85.86	84.66	84.50	84.58
vgg19	85.90	84.60	84.63	84.61
squeezenet	83.39	81.50	81.89	81.70
Inception V3	89.95	88.74	88.40	88.57
Google Net	81.32	79.62	79.41	79.51
Resnet50 L	85.89	83.95	83.94	83.95
Resnet50	93.41	92.59	92.55	92.57

**Table 5:** One-shot learning using 1-NN.

Features	Acc.	Pr	Re	F1
Hist L	4.58	4.92	4.63	4.77
Hist RGB	11.74	14.32	11.91	13.00
3 marg. hist.	13.20	15.28	13.45	14.30
qHist RGB	11.92	14.52	12.08	13.19
sHist RGB	7.06	9.42	7.27	8.20
Chrom. Mom.	7.31	7.65	7.42	7.54
Cooc. Matr.	1.69	1.90	1.69	1.79
SFTA	3.62	4.12	3.69	3.89
Granulometry	6.03	7.82	6.08	6.84
GIST	8.89	12.27	9.04	10.40
DT-CWT	7.86	9.43	7.98	8.65
CEDD	9.78	10.86	9.93	10.37
HOG	3.38	4.05	3.39	3.69
Gabor	7.43	8.97	7.52	8.18
LBP	6.49	8.46	6.51	7.36
LBP-nri	6.91	9.89	6.88	8.12
LBP-LCC	7.72	9.64	7.68	8.54
vgg16	27.70	32.86	27.94	30.20
vgg19	27.80	33.38	28.07	30.49
squeezenet	24.38	27.26	24.57	25.84
Inception V3	31.88	39.13	32.15	35.29
Google Net	24.17	29.12	24.35	26.52
Resnet50 L	30.21	36.21	30.45	33.08
Resnet50	43.48	50.55	43.83	46.95

## Data Availability

The images described in the paper and other ancillary information are available at the address http://www.ivl.disco.unimib.it/activities/t1k/.
